# A Novel Approach to the Room of Errors (ROE): A Three-Dimensional Virtual Tour Activity to Spotlight Patient Safety Threats

**DOI:** 10.7759/cureus.36130

**Published:** 2023-03-14

**Authors:** Kristen Mascarenhas, Marianfeli C Delgado Irahola, Alecia L Stein, Richard H Epstein, Roxanna Araya, Maureen Fitzpatrick, Joni M Maga

**Affiliations:** 1 Center for Patient Safety, Jackson Memorial Hospital / University of Miami Health System, Miami, USA; 2 Center for Patient Safety, Jackson Memorial Hospital / University of Miami, Miami, USA

**Keywords:** three-dimensional, patient safety, simulation, covid-19 pandemic, virtual reality, situational awareness

## Abstract

Background: Live simulation-based activities are effective tools in teaching situational awareness to improve patient safety training in healthcare settings. The coronavirus disease 2019 (COVID-19) pandemic forced the discontinuation of these in-person sessions. We describe our solution to this challenge: an online interactive activity titled the “Virtual Room of Errors.” The aim of this activity is to create an accessible and feasible method of educating healthcare providers about situational awareness in the hospital.

Materials and Methods: We applied existing three-dimensional virtual tour technology used in the real estate sector to a hospital patient room with a standardized patient and 46 intentionally placed hazards. Healthcare providers and students from our institution accessed the room online through a link where they independently navigate, and document observed safety hazards.

Results: In 2021 and 2022, a total of 510 learners completed the virtual Room of Errors (ROE). The virtual ROE increased annual participation in the activity, as compared to the in-person Room, and demonstrated learner satisfaction.

Conclusions: The virtual ROE is an accessible, feasible, and cost-effective method of educating healthcare workers on situational awareness of preventable hazards. Furthermore, the activity is a sustainable way to reach a larger number of learners from multiple disciplines, even as in-person activities resume.

## Introduction

Situational awareness has been defined as "conscious knowledge of the immediate environment and the events that are occurring in it” [1]. Failures to apply situational awareness in healthcare are intricately linked to medical errors and patient safety lapses [2]. As we will discuss, various methods, ranging from traditional tutorial lectures to interactive live-action simulations, have been employed to improve healthcare professionals’ level of situational awareness.

Simulation-based activity is the preferred modality employed in patient safety education, including those which focus on improving levels of situational awareness. Such training is highly targeted at improving patient outcomes and reducing medical errors in a healthcare environment [3]. An example is an activity in which learners physically move around a mocked-up hospital room and identify patient hazards, such as needles in the trash bin or water spills on the floor [4]. Pooled analysis of this simulation model from learners at multiple centers has shown that it is an effective and easily adaptable method to increase situational awareness for patient safety issues [4].

In the decade prior to the onset of the COVID-19 pandemic in 2020, our institution held a live “Room of Errors” event annually for staff, developed for National Patient Safety Awareness Week. The simulation activity was conducted within a patient room with a standardized patient. Learners were given a clipboard, paper, and pen. In the activity room, multiple learners worked independently to document any observed hazards. At the activity’s end, a member of the Center’s team met the learners to review their results. Learners had the opportunity to discuss their experience with the activity, including their own perception of challenges related to identifying hazards to patient safety within the Room. 

However, because of restrictions imposed by the onset of the COVID-19, the in-person Room of Errors (ROE) activity was no longer feasible. We decided to investigate options to translate our simulation to a virtual platform. Highly immersive virtual reality (VR) technologies have allowed for increased levels of learning customization and more precise healthcare environments [5]. VR has been used extensively to recreate the operating room environment to improve proficiency-based training, with studies showing that surgeons trained with VR are less likely to cause inadvertent injury [6]. While effective, these VR simulations tend to be expensive and not easily accessible because the technology is still relatively new. Healthcare education has also utilized 360-degree video technology, which is a less expensive, semi-immersive option. Studies have shown that it can be a viable alternative to VR, increasing learner information retention, attention, user satisfaction, and motivation [7-9]. However, a major limitation is that users are unable to independently navigate the environment as if they were physically in the room because the 360-degree videos are pre-recorded, and learners cannot control the display.

To overcome this limitation, our Center for Patient Safety (CPS) decided to engage a real estate media production company (Luxhunters Productions, Miami, FL), to create a user-friendly, immersive, interactive 3D virtual ROE. This employs the same technology used to create virtual home tours on real-estate sites such as Zillow® or realtor.com®. This technology improves 360-degree video technology by measuring space and adding 360-degree photos to a mesh that it creates from the dimensions, allowing the user to independently navigate through the composite images. We describe the process of developing our virtual ROE that we demonstrate to be low cost, high impact, and widely accessible to anyone with an internet connection. 

Also, portions of this research project were presented as a meeting abstract at the 2022 Florida Society of Anesthesiologist (FSA) Annual Meeting on June 11, 2022, and at the International Conference on the Future of Health Professions Education (ICFHPE) on November 3, 2022.

## Materials and methods

The University of Miami Institutional Review Board determined that this project, study # 20230142, was exempt from written participant consent by the learners.

Overview

In our virtual ROE, users independently navigate the room with over 44 hazards [see the table in the Appendix) that include clickable photos of pertinent patient care items (i.e., patient chart, identification band, medications, intravenous (IV) bag]. Planning and execution of the virtual Room of Errors took nine weeks (see the figure titled Planning and Execution of Virtual ROE in the Appendix). The virtual Room of Errors platform was launched during National Patient Safety Awareness Week in 2021 and was incorporated into the medical school patient safety curriculum later that year.

Equipment

We used a standard hospital patient room that is fully functional and contains typical equipment and supplies (i.e., patient bed, identification band, medications, IV bag). We used additional props such as yellow-colored water in a urinal and simulated blood. To create the interactive video, one-time use of 3D virtual tour photography equipment was required. The production company selected Matterport™ technology, Sunnyvale, CA, USA, a web-based platform to create a 3D digital representation of our Room of Errors. Accessing the virtual tour required a tablet, laptop, or desktop computer with internet access.

Personnel

We used a standardized patient, obtained from the UM Standardized Patient Center who verbally consented to the activity prior to being featured in the virtual room. The learners included medical students, nursing staff, environmental staff, and faculty members at our institution involved with patient care.

Creating the room

To determine which hazards to place in the room, we conducted literature searches of patient safety training activities, reviewed the Joint Commission 2021 National Patient Safety Goals [10], and numerous healthcare professionals from various specialties and departments collaborated (Table 1).

**Table 1:** Sample room hazards and National Patient Safety Goals 2021. NPSG, National Patient Safety Goals; IV, intravenous

Room hazards	NPSG	Goal defined
The wrong patient's name appears inside the chart.	NPSG.01.01.01	Use at least two ways to identify patients.
IV bag expired in 2016, neither drip rate nor dosage on IV bag.	NPSG.03.06.01	Maintain and communicate accurate patient medication information.
The hand disinfectant dispenser is empty.	NPSG.07.01.01	Use the hand cleaning guidelines from the Centers for Disease Control and Prevention or the World Health Organization.
Bloody gauze is left on the trash bin and is accessible to the patient and his visitors.	NPSG.07.01.01	Use the hand cleaning guidelines from the Centers for Disease Control and Prevention or the World Health Organization.
Bloody gloves left accessible to the patient and his visitors.	NPSG.07.01.01	Use the hand cleaning guidelines from the Centers for Disease Control and Prevention or the World Health Organization.
Cleaning detergents are accessible next to the shower.	NPSG.15.01.01	Reduce the risk of suicide.
Curtain roller blind chain is disassembled.	NPSG.15.01.01	Reduce the risk of suicide.
Exposed sharps are accessible.	NPSG.15.01.01	Reduce the risk of suicide.
Lengthy telephone cord.	NPSG.15.01.01	Reduce the risk of suicide.
Scissors and a razor are accessible.	NPSG.15.01.01	Reduce the risk of suicide.

Planned hazards were categorized as those pertaining to patient, environment, equipment, medications, and care process. Patient hazards were defined as any threat which is in direct contact with the patient. Environmental hazards were those which could potentially cause harm to a patient or any person walking into the room. Equipment hazards were those related to medical equipment. Medication-related hazards were improper labeling, expired medications, and/or medications left unattended. Care processes were established as any hazards caused by the systems of care (i.e., two different names on the patient’s chart). All names, medical record numbers, and financial identification numbers were fabricated.

Implementation

The hospital provided an unoccupied semi-private room in a medical-surgical unit. This choice allowed ample spatial movement during the activity and the ability to spread the hazards liberally throughout space. The room was 14 feet x 18 feet (4.27 m x 5.49 m), and each patient space included a bed, tray table, IV pole, privacy curtain, and bedside table. The shared restroom was 4 feet x 7 feet (1.22 m x 2.13 m) and included standard features plus a laundry bin and trash can. Forty-five minutes were required to set up the hazards throughout the patient room. The standardized patient was briefed on the impending videographic activity for 3 min. Then, he received a patient’s gown, nasal cannula, identification wristband, allergy wristband, simulated IV bag, simulated urinary catheter tubing, and a gauze bandage (soaked with simulated blood) applied to the simulated left knee surgical site. Half an hour later, we met with the videographer to finalize the room elements, discuss expectations, and allow for Matterport technology to capture the room details and digitize three separate images (identification wristband, patient chart, and IV bag) that would become embedded in the 3D virtual room. After filming was completed, the props were collected by the Center’s team and housekeeping was contacted to clean the room for actual patient use.

Our production cost was approximately $300, but we estimate that this could be up to $1000 as an initial expense for the development of the virtual room, depending on the extent to which various hazards and props might need to be created. Our annual maintenance hosting fee is approximately $60 but, in Miami, could range up to $100. The rate for a standardized patient for 4 h in Miami ranges from $50 to $150 and our cost was $54. Permission was granted from the production company and the standardized patient for purposes of publication.

The link (https://my.matterport.com/show/?m=s6GHso1dte7) to the activity was either emailed to learners or shared via electronic communications such as social media posts and email campaigns. Learners received instructions which detailed the tour of a simulated clinical environment that is filled with patient safety hazards and included the activity objectives.

The activity

**Figure 1:**
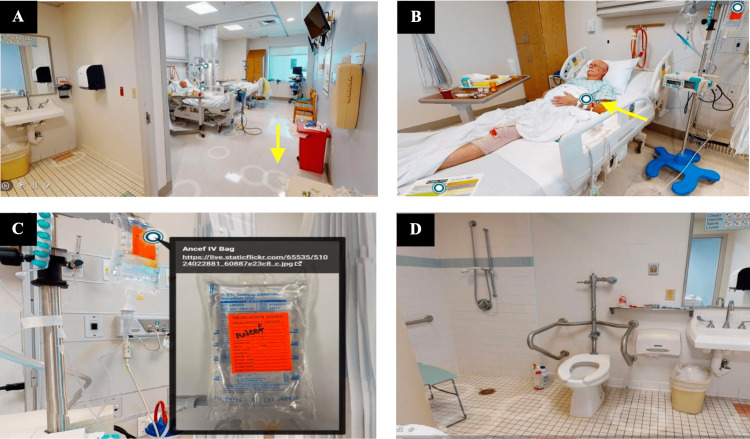
Virtual ROE. A: The white circles (yellow arrow) represent clickable elements to allow navigation throughout the rooms. B: The blue/white circle (yellow arrow) represents embedded photos that can be clicked for closer details. C: Embedded photo of antibiotic bag that enlarges when blue/white circle is clicked. D: Patient bathroom. ROE, Room of Errors

The module begins with movement from a brief title page introduction to a 3D aerial dollhouse view of the expansive patient room floorplan. The image then glides the learner to the floor level and places the learner just beyond the room entrance providing the perspective of standing inside the room. White circles placed throughout the virtual room are clickable elements that allow navigation throughout the environment (Figure 1A). Blue and white circles that are displayed represent embedded photos (i.e., the patient’s identification wristband, IV antibiotic bag, patient’s chart), as seen in Figure 1B,C. Also depicted is the patient bathroom (Figure 1D).

The standardized patient is resting in the bed nearest to the door. Also within the view are visitor chairs, a sharps container, a whiteboard, equipment, and a window at the furthest end of the room. A full list of identifiable hazards is provided in the table in the Appendix. There was no time limit applied to complete the activity.

Learners documented the hazards they identified and completed an evaluation (see the figure titled Virtual ROE Entry Form in the Appendix) of the simulation experience via JotForm (JotForm, Inc., San Francisco, CA). The evaluation included a series of five-point Likert scale statements as well as a free-text comment section to assess user engagement and comfort level with the activity. Following completion of the activity and survey submission, learners received a copy of their hazard submission form via an automated email programmed into JotForm, which included the identified hazards, survey responses, and a link to the complete list of hazards. In a later iteration, learners had the opportunity to discuss further and ask questions regarding the simulation activity with a designated contact person from the CPS via Zoom (Zoom Video Communications, San Jose, CA).

## Results

A total of 510 learners completed the virtual ROE activity in 2021 and 2022. The virtual ROE allowed for increased learner access and participation, as reflected in Table 2. The number of learners increased from the in-person room in 2019 to the virtual room in 2021 and 2022. Participation did drop from 2021 to 2022, but this decrease was due to the widespread introduction of the activity to all learners in 2021, so that by 2022, there were fewer new learners who had not yet completed the activity.

**Table 2:** ROE participation and hazard identification. ROE, Room of Error

Measurements	2019 (in-person)	2021 (virtual)	2022 (virtual)
Number of participants	89	341	169
Mean hazards identified +/- SD	11.9 +/- 3.7	19.2 +/- 12.0	15.3 +/- 10.1
Median hazards identified (interquartile range)	11 (10 to 14)	17 (11 to 25)	13 (9 to 19)
Mode hazards identified	10	12	10

Results of the Likert scale questionnaire are found in Table 3. Over 74% of learners “strongly agreed” or “agreed” that the activity increased their comfort level to navigate a 3D virtual simulation-based platform, while 80% of learners “strongly agreed” or “agreed” that this activity improved their situational awareness to identify hazards that can be present within a patient room.

**Table 3:** Five-point Likert scale evaluation of the virtual ROE. ROE, Room of Errors

Statement	1 = Strongly Disagree	2 = Disagree	3 = Neutral	4 = Agree	5 = Strongly Agree	Average Response (1-5) +/- SD
The objectives of the 3D virtual ROE activity were clear to me	3 (1.8%)	4 (2.4%)	22 (13.4%)	64 (39.0%)	70 (42.7%)	4.2 +/- 0.9
It was a worthwhile learning experience	4 (2.4%)	10 (6.1%)	28 (17.1%)	62 (37.8%)	59 (36.0%)	4.0 +/- 1.0
This activity increased my comfort level to navigate a 3D virtual simulation-based platform	4 (2.4%)	5 (3.0%)	32 (10.5%)	64 (39.0%)	58 (35.4%)	4.0 +/- 1.0
I was able to demonstrate my ability to recognize patient safety hazards and provide examples	2 (1.2%)	5 (3.0%)	26 (25.8%)	87 (53.0%)	43 (26.2%)	4.01 +/- 0.8
This activity improved my situational awareness to identify hazards that can be present within a patient room	2 (1.2%)	3 (1.8%)	27 (16.5%)	80 (48.8%)	51 (31.1%)	4.1 +/- 0.8

Forty learners chose to provide feedback through free-text comments at the end of the survey. Of these responses, 24 were coded as “Positive,” eight were coded as “Neutral,” and eight were coded as “Negative.” Users provided positive qualitative feedback regarding their own perception of their situational awareness, which included, “I think the exercise is a good method of getting us in the practice of scanning each room for errors front to back when going to meet our patients.” One learner noted that the activity “felt fun as well as educational, which is a nice change from some of the less interactive assignments,” demonstrating improved learner satisfaction. Negative feedback included a user noting, “It was hard to look closely at details.” Neutral feedback included multiple recommendations to have a “list of certain errors that we should look for beforehand to have a better idea of what to keep an eye out for.” 

Some learners also commented on the efficacy of the online version of the activity: “The ability to travel virtually and navigate the environment via many angles using the program was pretty cool. I appreciated the functionality of viewing the patient chart and to get a better visualization of the IV pump, the patient wristband, etc.”

## Discussion

Although our initiative to create a virtual simulation experience for learners to identify hospital room hazards was borne from the COVID-19 pandemic, we quickly realized that the virtual offering allowed us to reach far more users than we were able to in the past. For over a decade, annual in-person ROE participation was limited to those learners who were at work on a day that the ROE was set up, whose work assignments were physically located near the room, and to those who had a break in their schedule that coincided with the room’s availability. However, with the virtual ROE, we now had the opportunity to provide limitless participation as the educational offering was now no longer constrained by any space, time, availability, or schedule. These findings are consistent with a study by Hofstädter-Thalmann et al., concluding that virtual learning is a valid alternative to face-to-face conferences and can attract a wider audience including those non-traditional learners and off-site learners [11]. 

Although participation decreased between 2021 and 2022 (Table 2), we attribute this to the fact that the activity was widely introduced as a one-time practice of situational awareness in this 3D virtual platform. We anticipate this being an ongoing activity, with learners repeating the exercise every few years, with future iterations to ensure providers are frequently reminded of safety precautions within the hospital. 

The process of developing and implementing our virtual ROE was straightforward and inexpensive. We were able to set up the room and complete filming within a single day, which contributed to the cost-effectiveness and simplicity of our model, making this tool easily replicable in a variety of disciplines and at other institutions. Satnarine and Lee Kin also concluded similar advantages in virtual medical student rotations such as cost and time savings, increased flexibility of the offerings, as well as increased participation [12].

Quantitative feedback obtained was encouraging (Table 3), as it indicated comfort navigating a 3D virtual simulation platform as well as subjective improved situational awareness through the activity. The qualitative feedback gathered also highlights the unique ability of this training tool to allow the user to independently explore the room. This is consistent with the positive feedback obtained from participants of virtual medical training published by De Ponti et al. In addition, the participants considered the future use of this virtual training useful in concert with traditional bedside training [13]. This positive feedback also aligns with that observed in a study by Alharbi et al. comparing in-person to virtual anatomy lab dissection learning from medical students [14].

One limitation of implementing this activity included the availability and gaining access to the hospital room to set up the activity. Another potential barrier to consider is the approvals required for the videographer to gain access and film a hospital room. Finding a volunteer or standardized patient willing to portray the patient and be filmed is another potential obstacle. Additionally, access to props might be a potential barrier. At our institution, the CPS has a set of supplies we were able to use in setting up our room, but other institutions might need to purchase or build props. Although learners are able to interact with the room, the room is not able to interact back with the learners. This decision arose from our goal of creating a cost-effective activity that is easy to implement at other institutions, though this does make our activity less interactive compared to other more expensive simulation activities available [15]. Another limitation in our approach was that we did not have an independent reviewer code for the free-text responses in the feedback survey. This addition would have removed potential bias in the qualitative feedback data analysis.

Due to the variety of responses and missed hazards, we incorporated a formal debriefing over Zoom in a later iteration. We believe this to be beneficial in reemphasizing concepts around situational awareness, as suggested by Zimmermann et al. and Niu et al., who emphasized the importance and benefit of the debriefing after the learning activity to enhance interprofessional trainings and education [4, 16]. In addition, a formal debrief allows for discussion about how these hazards can be prevented in the clinical environment. We visualize many potential applications for the virtual ROE: the operating room, emergency department, outpatient clinic, physical therapy clinics, laboratories, etc. The activity has low resource requirements and business providing 3D virtual videography are widely available, given the ubiquitous presence of virtual tours in the real estate domain. Thus, our described implementation should easily be replicable in other hospital systems. We have already begun our second iteration of the ROE within our hospital’s Pediatric Infection Disease Department.

## Conclusions

During the COVID-19 pandemic, our mainstay of teaching situational awareness with regards to identification of patient room hazards was blocked by hospital access and social distancing restrictions. The 3D virtual real estate tour technology allowed us to inexpensively migrate this activity to an online platform, which expanded participation through improved accessibility. Despite most COVID-19 restrictions now being lifted at our hospital, we plan to continue to develop additional virtual Rooms of Errors, as it successfully addresses our goals of teaching situational awareness in a feasible, accessible, and cost-effective manner. Other institutions can benefit from our experiences in setting up similar virtual environments to teach situational awareness in a variety of scenarios.

## Figures and Tables

**Figure 2:**
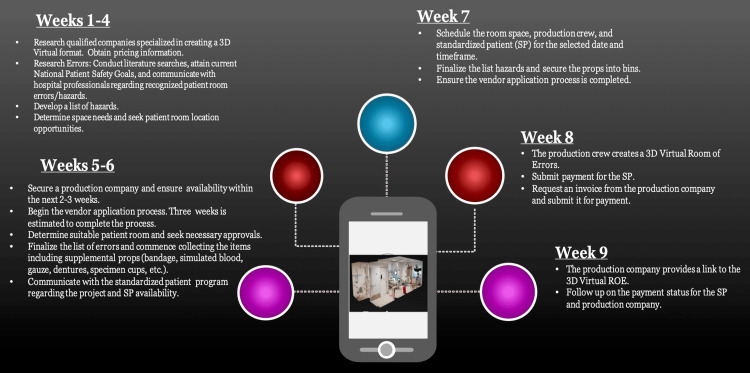
Planning and execution of virtual ROE. ROE, Room of Errors

**Figure 3:**
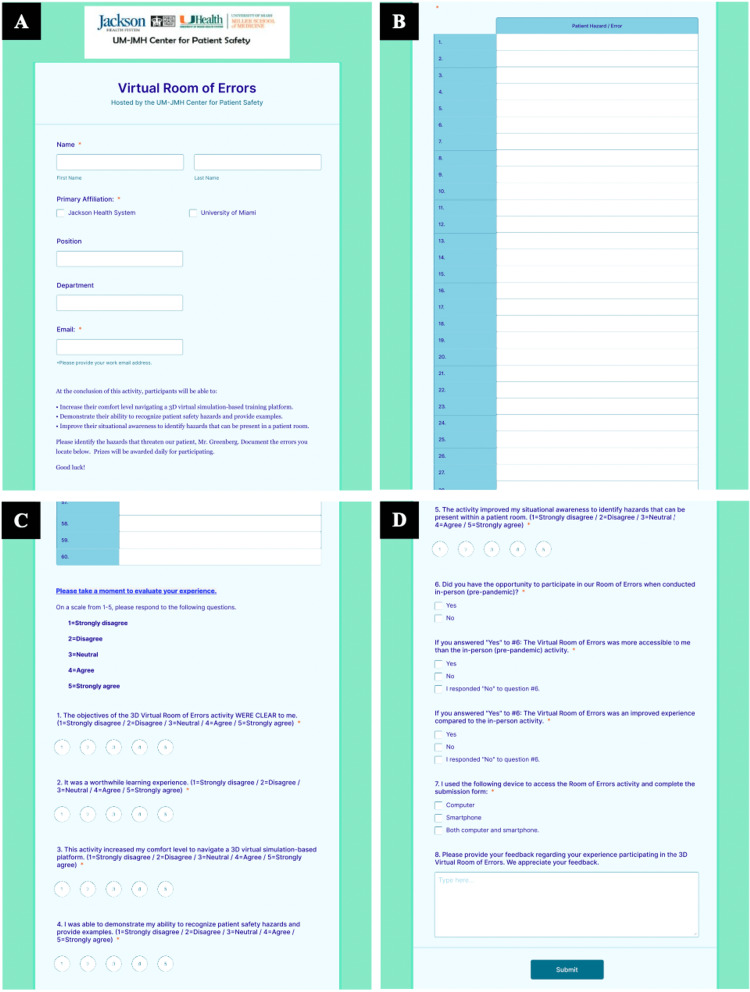
Virtual ROE entry form. ROE, Room of Errors

## Appendices

**Table 4:** List of hazards. WOW, workstation on wheels; QD, every day; DVT, deep vein thrombosis; IV, intravenous

	Area category	Hazard
1	Bathroom	Soap dispenser is empty
2	Bathroom	Clean linen on top of dirty laundry hamper
3	Bathroom	Scissors, used antiseptic, razor by handwashing sink
4	Bathroom	Paper towel dispenser empty
5	Bathroom	Cleaning detergents next to shower
6	Bathroom	Specimen cups above soap dispenser
7	Bathroom	Soiled gauzed on safety bar and dirty towels
8	Biohazard	Bin overflowing, soiled gloves, gauze and trash on top
9	Fall risk	Patient bed in high position
10	Fall risk	Falls risk yellow bracelet in hanging from the pole
11	Fall risk	Yellow socks draped on footboard
12	Fall Risk	Bedsides rails are down
13	Fall risk	Water spill on the floor
14	IV pole	Oral suction tip and oral care products not covered
15	IV pole	Used Disinfecting port protectors left over
16	IV pole	IV bag expired 2016, neither drip rate nor dosage on IV bag
17	IV pole	Syringe with clear fluid is connected to the IV bag
18	IV pole	IV bag empty
19	Overbed table	Used urinal near foot items, medications, and water
20	Overbed table	Partial dentures on overbed table
21	Overbed table	Medications left unattended and sharps within reach
22	Patient	Foley catheter tubing wrapped through bed rails
23	Patient	Call button out of reach
24	Patient	Bed is disconnected
25	Patient	Nasal cannula/oxygen is on patient forehead
26	Patient	DVT compression pump is not connected to the patient and cords dragging along the floor
27	Patient	Left knee dressing is soiled
28	Patient chart	Wrong patient name (fabricated) appears on bottom chart
29	Patient chart	Surgery site is on right knee and the left knee is bandaged
30	Patient chart	Incorrect abbreviations (e.g., QD instead of daily)
31	Patient chart	Using trailing zeroes (X.0mg), lack of leading zero (.x mg)
32	Patient chart	It says male patient is in third trimester of pregnancy
33	Patient room	Purell dispenser empty
34	Patient room	IV bag and soiled gauze left on top of trash bin
35	Patient room	Trash container overflowing
36	Patient room	Used isolation gown left out
37	Sharps	Exposed sharps on top of pulse oximeter sensors bin
38	Sharps	Exposed sharps on tip of sharps bin
39	Whiteboard	Incorrect dates and incomplete information
40	Windowsill	Phone cord stretching across room
41	Windowsill	Syringes, needles, scissors, and curtain is unhooked
42	WOW	Staff personal belongings in the clear bag hanging on it
43	WOW	Unplugged
44	WOW	Needles, dirty gloves, unlabeled tubes
